# Supplementary material for: Particle-associated denitrification is the primary source of N2O in oxic coastal waters
Source: Nat Commun. 2023 Dec 13;14:8280. doi: 10.1038/s41467-023-43997-3 (PMC10719265; doi:10.1038/s41467-023-43997-3)
Supplement: Supplementary file 1 — Supplementary Information [file 41467_2023_43997_MOESM1_ESM.pdf]

Supplementary Information:

Particle-associated denitrification is the primary source of N_2O in oxic coastal waters

Xianhui S. Wan[1, 2*], Hua-Xia Sheng[1], Li Liu[1], Hui Shen[1], Weiyi Tang[2], Wenbin Zou[1], Min N. Xu[3], Zhenzhen Zheng[3], Ehui Tan[3], Mingming Chen[1], Yao Zhang[1], Bess B. Ward[2] & Shuh-Ji Kao[1, 3*]

[1] College of Ocean and Earth Science, State Key Laboratory of Marine Environmental Sciences, Xiamen University, Xiamen 361101, China

[2] Department of Geosciences, Princeton University, NJ 08540, USA

[3] State Key Laboratory of Marine Resource Utilization in South China Sea, Hainan University, Haikou, 570208, China

[*] Corresponding authors: Xianhui S. Wan; Shuh-Ji Kao

Email: xianhuiw@princeton.edu; sjkao@xmu.edu.cn

This file includes:

Supplementary Figures 1-11;

Supplementary Tables 1-5;

Supplementary Notes 1-2;

Supplementary References.

Supplementary Figures

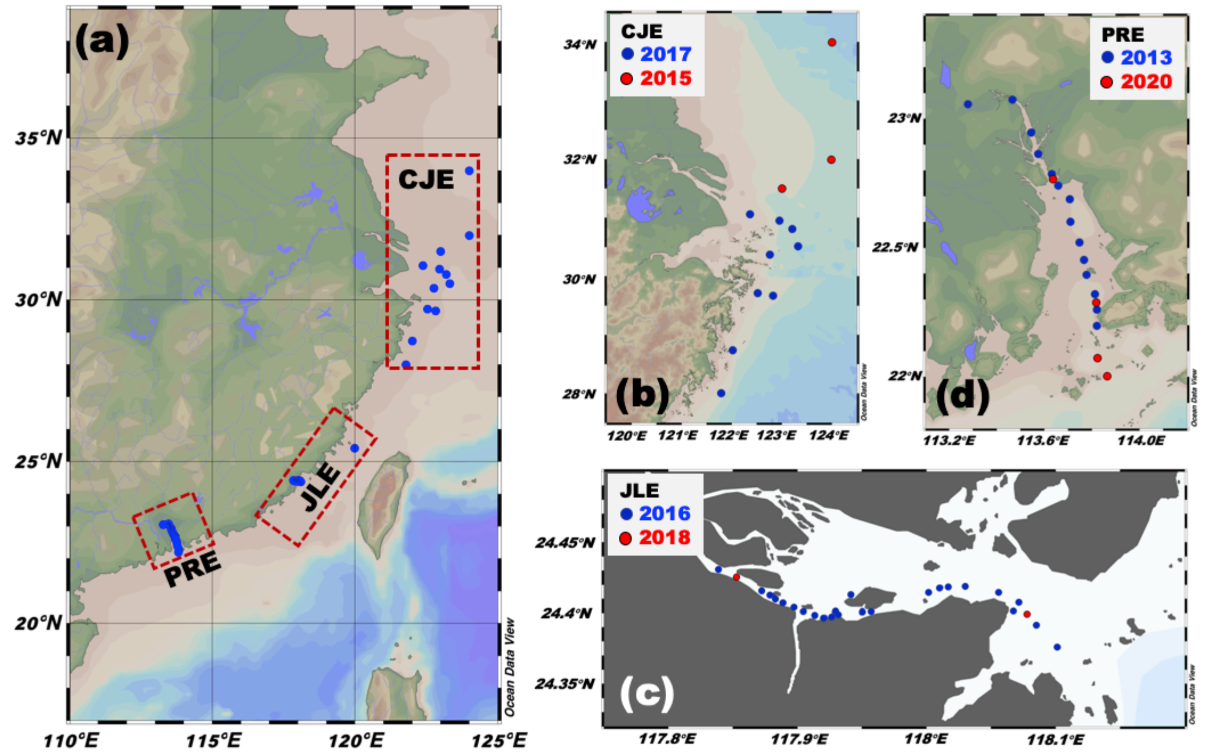

Supplementary Fig. 1 Study area and sampling stations. a: overview of the research area; b-d: sampling stations distribution at each cruise. CJE, JLE, PRE were acronyms of the Changjiang Estuary and its adjacent East China Sea shelf (b), the Jiulong Estuary and its adjacent Taiwan Strait (c), and the Pearl River Estuary and its adjacent South China Sea shelf (d), respectively. The figure was created by using Ocean Data View 4.6.3 (http://odv.awi.de)[1].

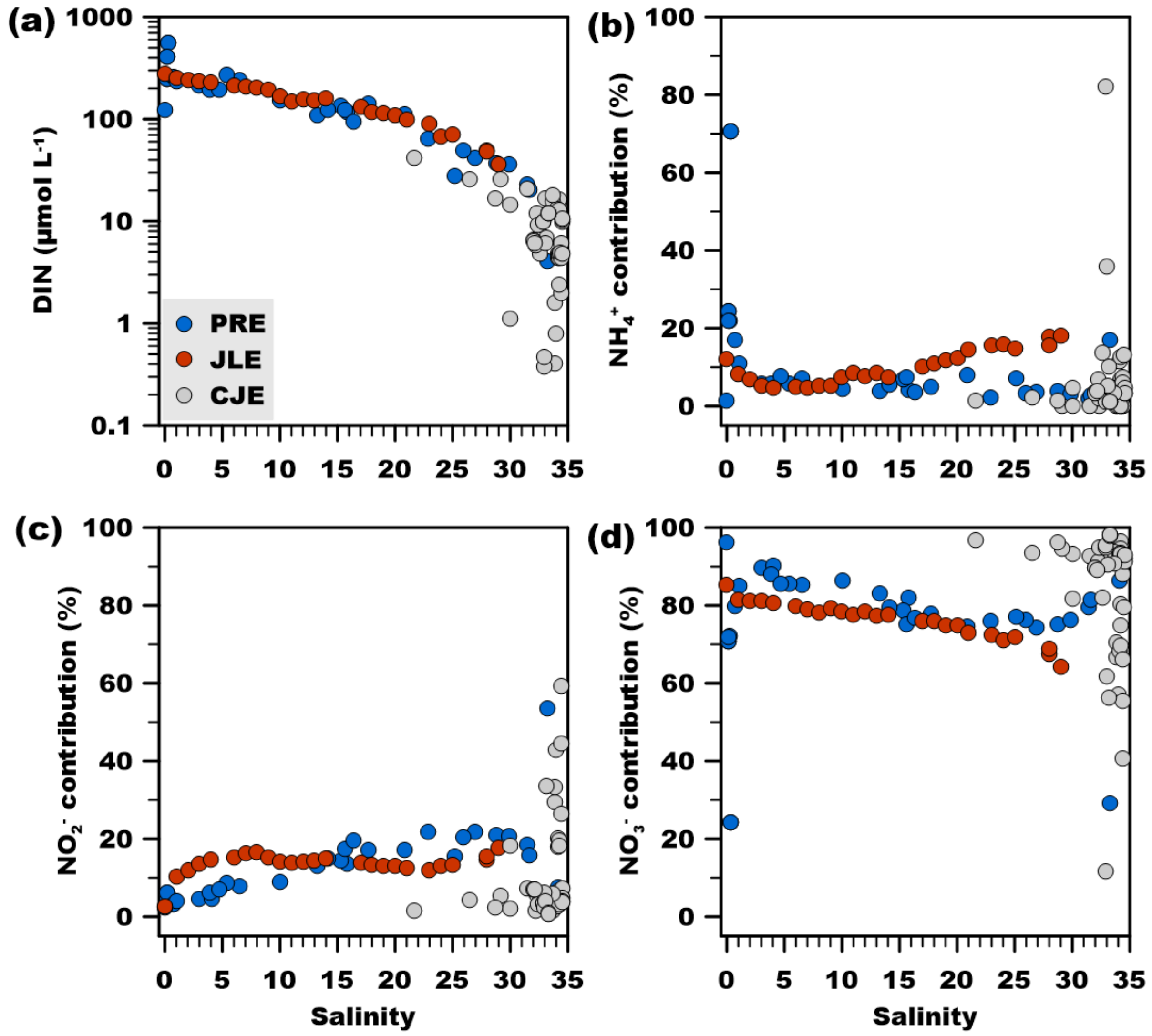

Supplementary Fig. 2 Concentration and composition of DIN. a: DIN concentration; b-d: fractional contribution of NH_4^+, NO_2^-, and NO_3^- to the DIN pool, respectively. The blue, red and gray dots denote samples from the PRE (2013 and 2020), JLE (2016 and 2018) and CJE (2015 and 2017), respectively.

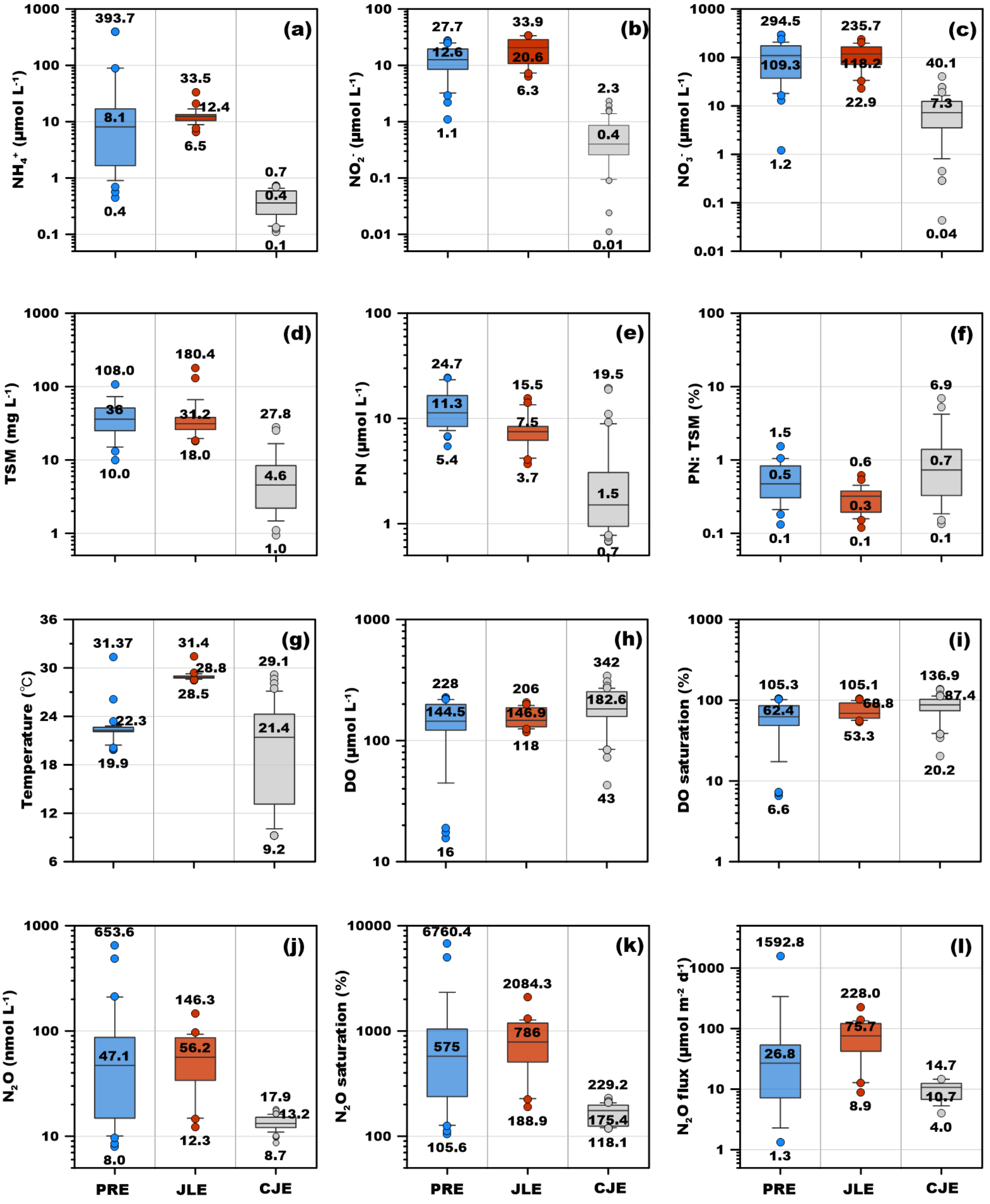

Supplementary Fig. 3 Statistics of nutrient, DO and N_2O distributions. a: NH_4^+; b: NO_2^-, c: NO_3^-; d: TSM; e: PN; f: PN: TSM; g: temperature; h: DO; i: DO saturation; j: N_2O concentration; k: N_2O saturation; l: N_2O flux. The blue, red and gray dots denote samples from the PRE (2013 and 2020), JLE (2016 and 2018) and CJE (2015 and 2017), respectively. The numbers in the box plots show the median value, and the minima and maxima values, respectively; whiskers and boxes show the 10% and 90% percentile and 25-75% quartile of the measurements, respectively.

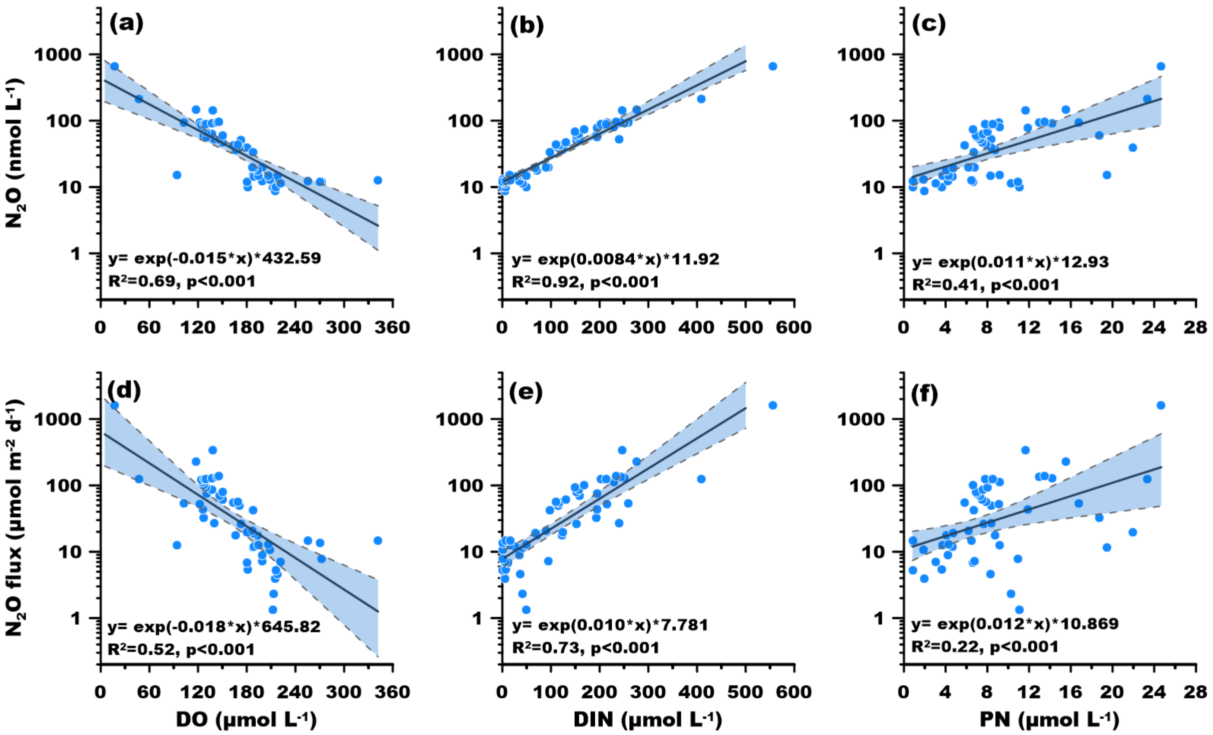

Supplementary Fig. 4 Relationships between N_2O, N_2O flux and DO, DIN and PN concentrations. The relationships were best fitted by exponential regression. The black lines and gray shadows show linear regressions and the 95 % confidence intervals, respectively.

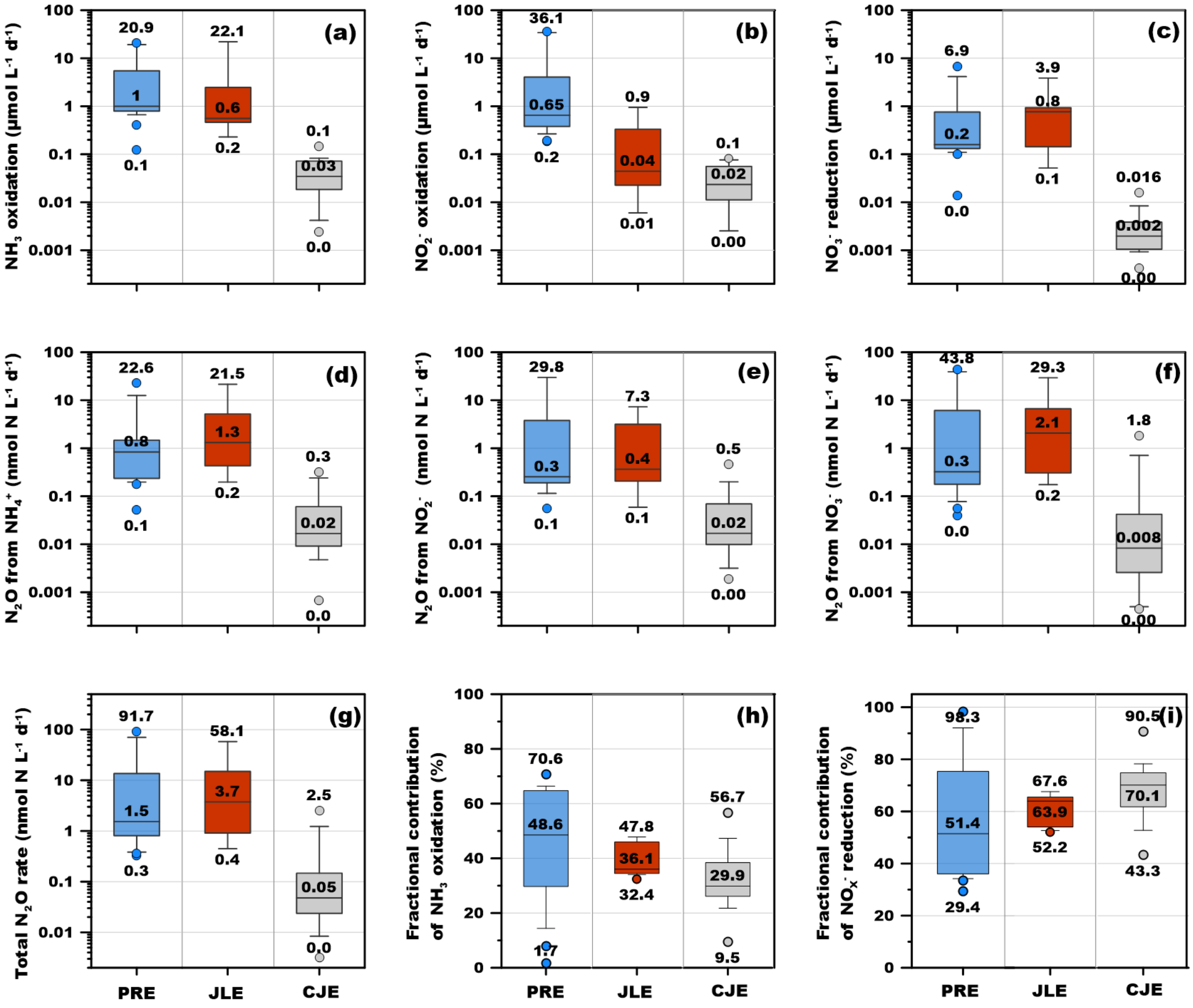

Supplementary Fig. 5 Statistics of N conversion rates and N_2O production rates from multiple substrates. a: ammonia oxidation; b: NO_2^- oxidation; c: NO_3^- reduction to NO_2^-; d: N_2O production from NH_4^+; e: N_2O production from NO_2^-; f: N_2O production from NO_3^-; g: total N_2O production rate; h: fractional contribution of N_2O production from ammonia oxidation; i: fractional contribution of N_2O production from NO_X^- reduction. The blue, red and gray dots denote samples from the PRE, JLE and CJE, respectively. The numbers in the box plots show the median value, and the minima and maxima values, respectively; whiskers and boxes show the 10% and 90% percentile and 25-75% quartile of the measurements, respectively (n = 20, 9, 19 stations in the PRE, JLE, and CJE, respectively).

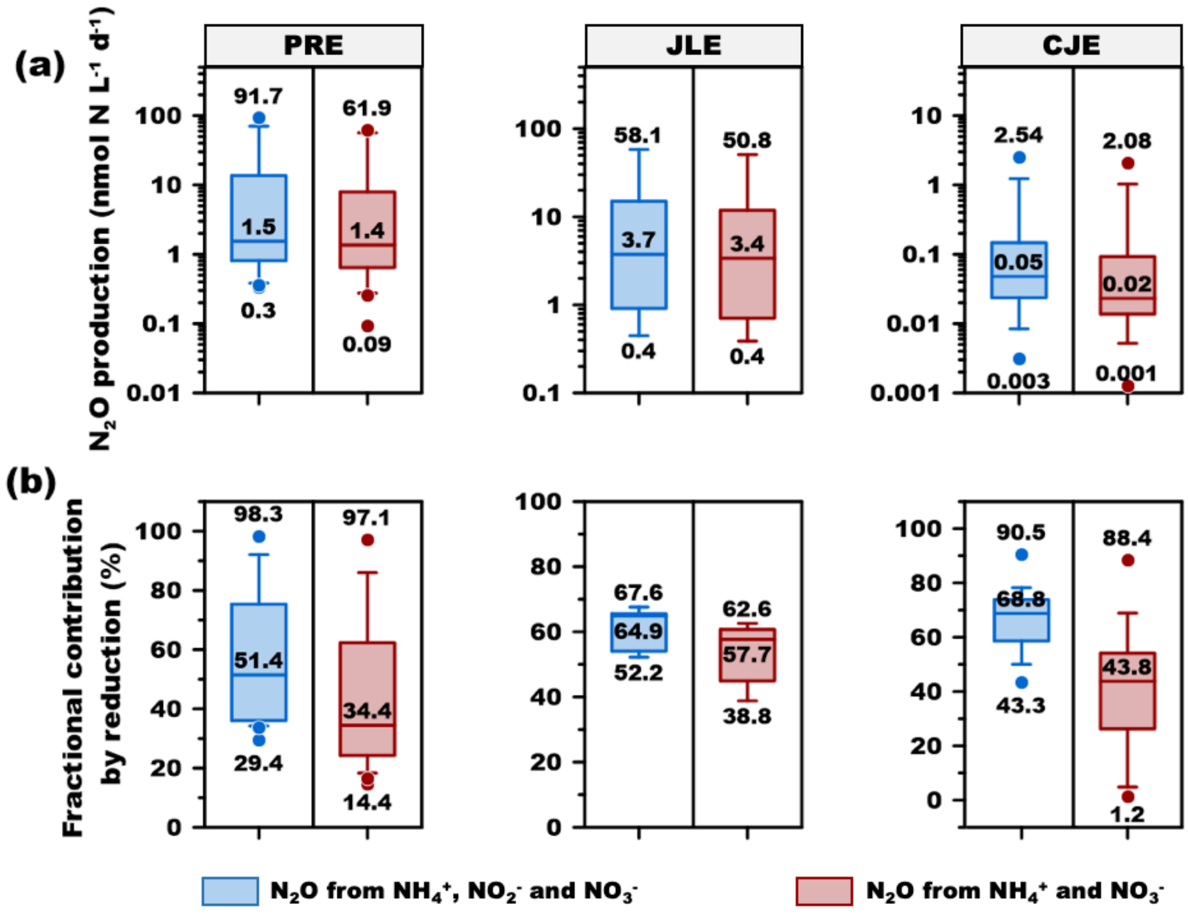

Supplementary Fig. 6 Comparison of N_2O production by oxidative and reductive pathways. a: total N_2O production rate; b: fraction of total N_2O production due to the reductive pathway considering results from all tracers ($^{15}NH_4^+$, $^{15}NO_2^-$ and $^{15}NO_3^-$) (blue bars) or from $^{15}NH_4^+$ and $^{15}NO_3^-$ only (i.e., removing N_2O production from $^{15}NO_2^-$) (red bars). The numbers in the box plots show the median value, and the minima and maxima values, respectively; whiskers and boxes show the 10% and 90% percentile and 25-75% quartile of the measurements, respectively.

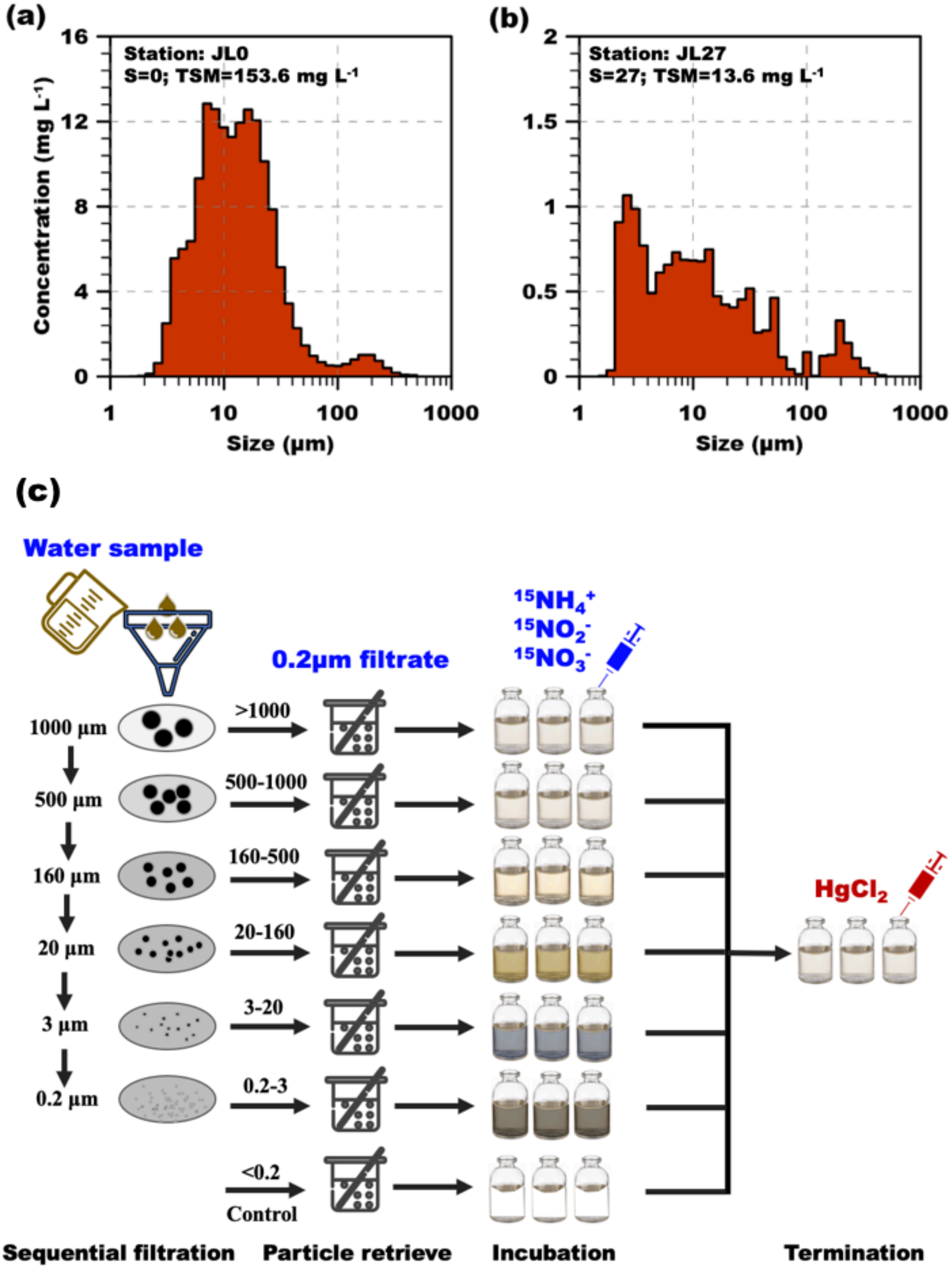

Supplementary Fig. 7 Size structure of TSM and schematic for the sequential size-fractionated incubation at the JLE (2018). a, b: size structure and concentration of TSM at the upstream (JL0) and downstream (JL27) estuarine stations. c: schematic of the size-fractionated manipulation process.

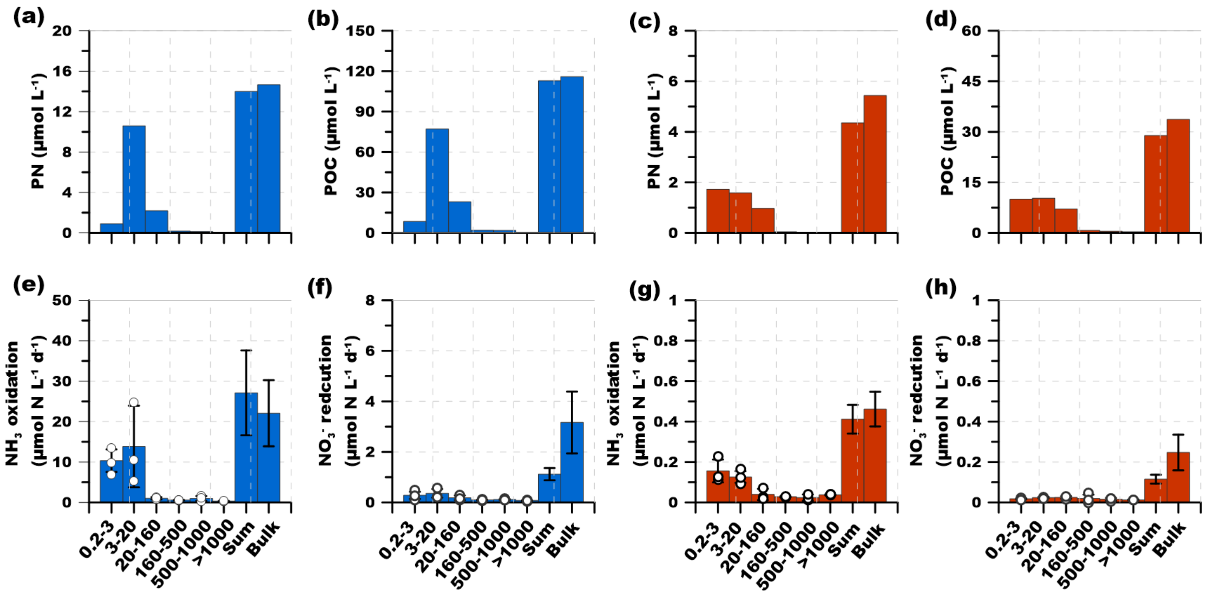

Supplementary Fig. 8 Recovery efficiency of POM and N conversion rate of size-fractionated filtration at two stations in the JLE (2018). a-d: recovery efficiency of PN and POC; e-h: recovery efficiency of ammonia oxidation and NO_3^- reduction. The blue and red bars show the results from upstream station JL0 (Salinity=0) and downstream station JL27 (Salinity=27), respectively. Data are presented as mean rates ± standard deviation. Errors bars are standard deviation from triplicates incubation (*n* = 3 biologically independent samples). Note there were no replicates for PN and POC concentration measurement.

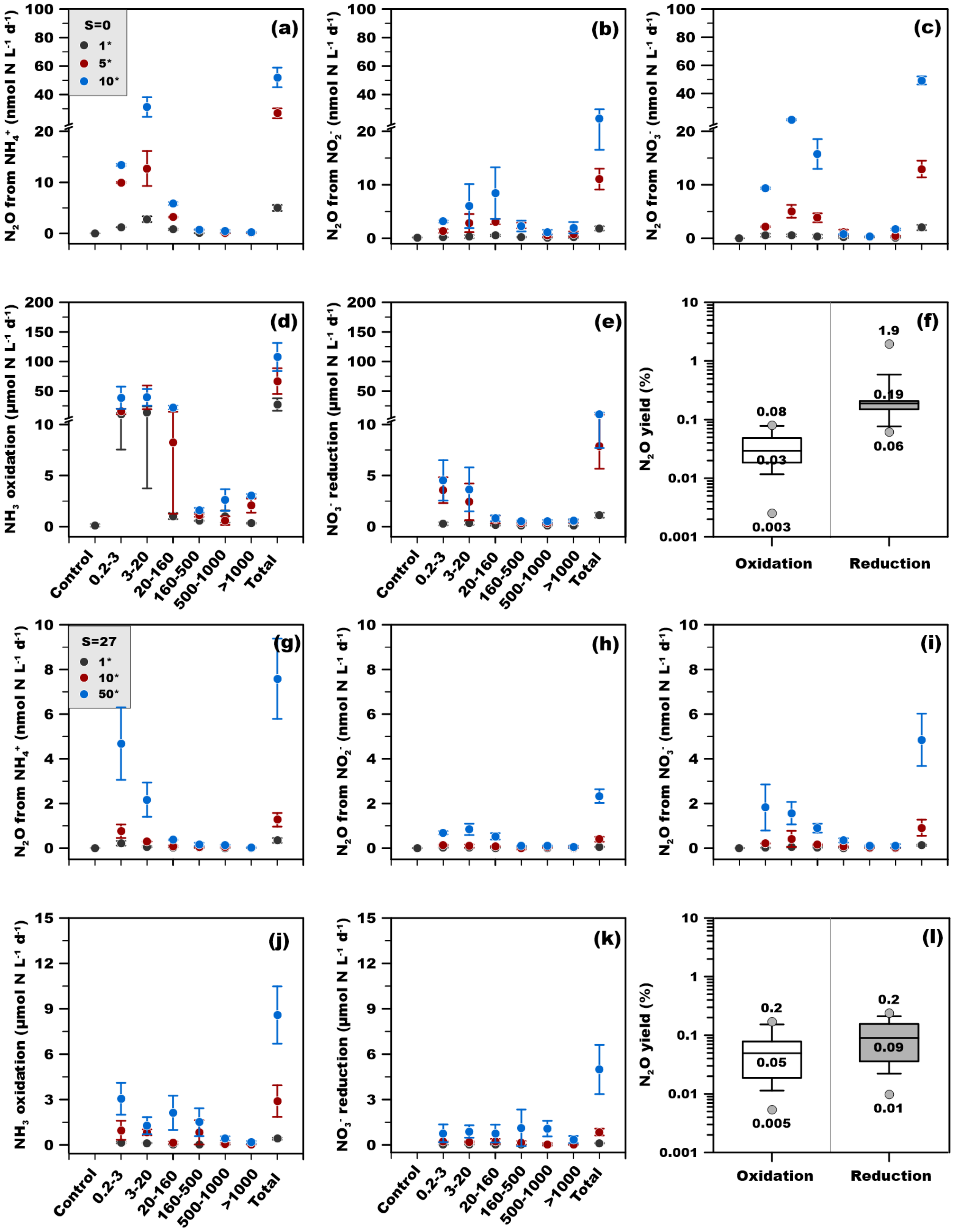

Supplementary Fig. 9 Size-fractionated N conversion and N_2O production rates in the JLE (2018). a-f: rates measured at upstream station JL0 (salinity=0), g-l: rates measured at downstream station JL27 (salinity=27). a, d: N_2O from NH_4^+, b, h: N_2O from NO_2^-, c, i: N_2O from NO_3^-; d, j: ammonia oxidation, e, k: NO_3^- reduction to NO_2^-, f, l: N_2O yield. The gray, red

and blue dots denote rates derived from incubation with particle enrichment of 1, 5, and 10-fold compared to in-situ concentration at the upstream station in panels a-e; and represent rates derived from incubation with particle enrichment of 1, 10, and 50-fold compared to in-situ concentration in the lower station in panels g-k. At each station, six sizes of particles are retrieved by sequential filtration. Data are presented as mean rates ± standard deviation. Errors bars are standard deviation from triplicates incubation (*n* = 3 biologically independent samples). The white and gray bars in panels f and l show N_2O yield during ammonia oxidation and NO_3^- reduction. The numbers in the box plots show the median value, and the minima and maxima values, respectively; whiskers and boxes show the 10% and 90% percentile and 25-75% quartile of the measurements, respectively.

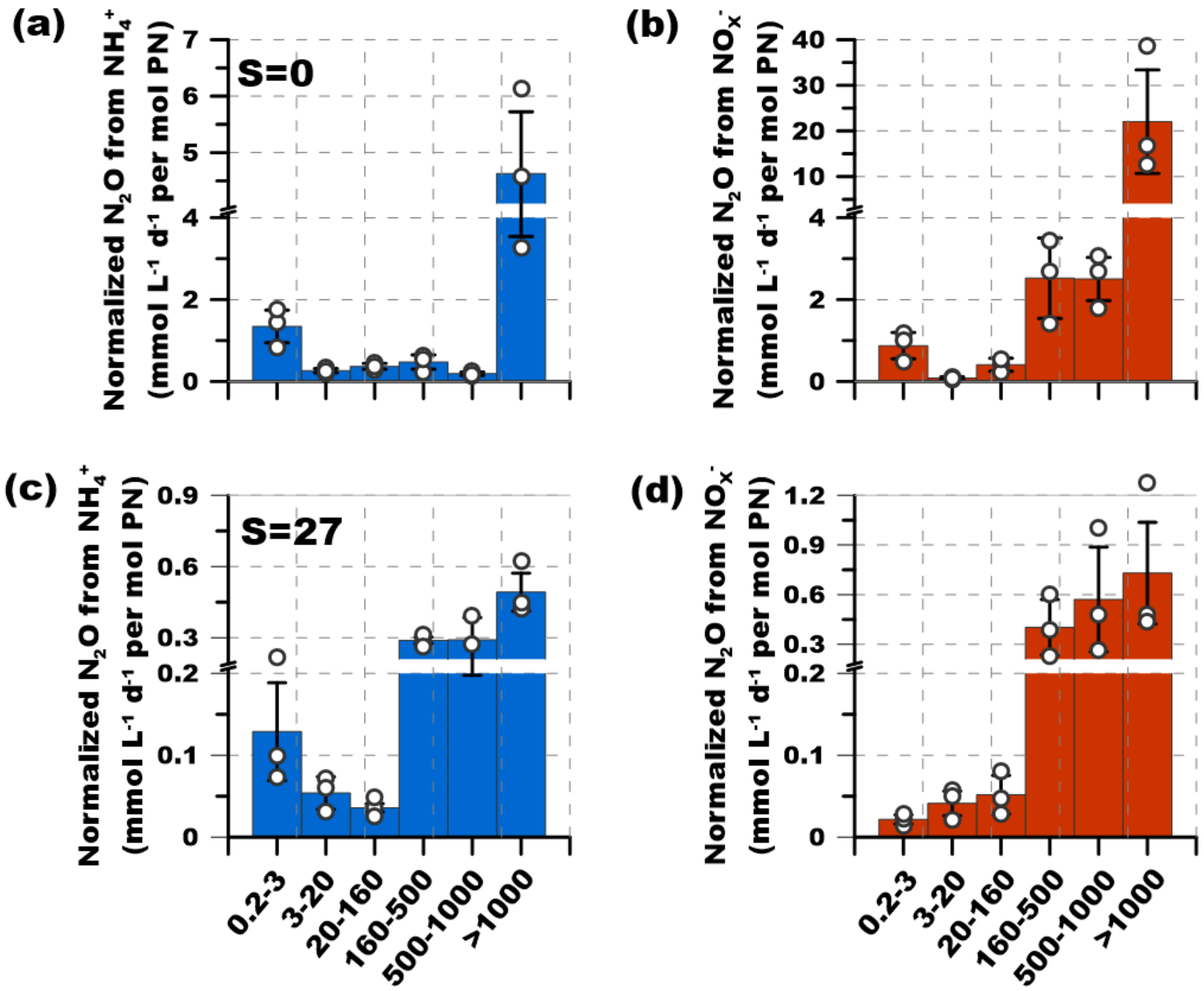

Supplementary Fig. 10 Normalized size-fractionated N_2O production rates in the JLE (2018). The rates are normalized to per mol PN at each size. a-b: rates measured at upstream station JL0 (salinity=0); c-d: rates measured at upstream station JL27 (salinity=27). a, c: N_2O from NH_4^+; b, d: N_2O from NO_X^-. At each station, six sizes of particles are retrieved by sequential filtration. Data are presented as mean rates ± standard deviation. Errors bars are standard deviation from triplicates incubation (*n* = 3 biologically independent samples). Note that the normalization was only performed in the 1-fold particle retrieval manipulation, because size-fractionated PN concentration was measured in this group.

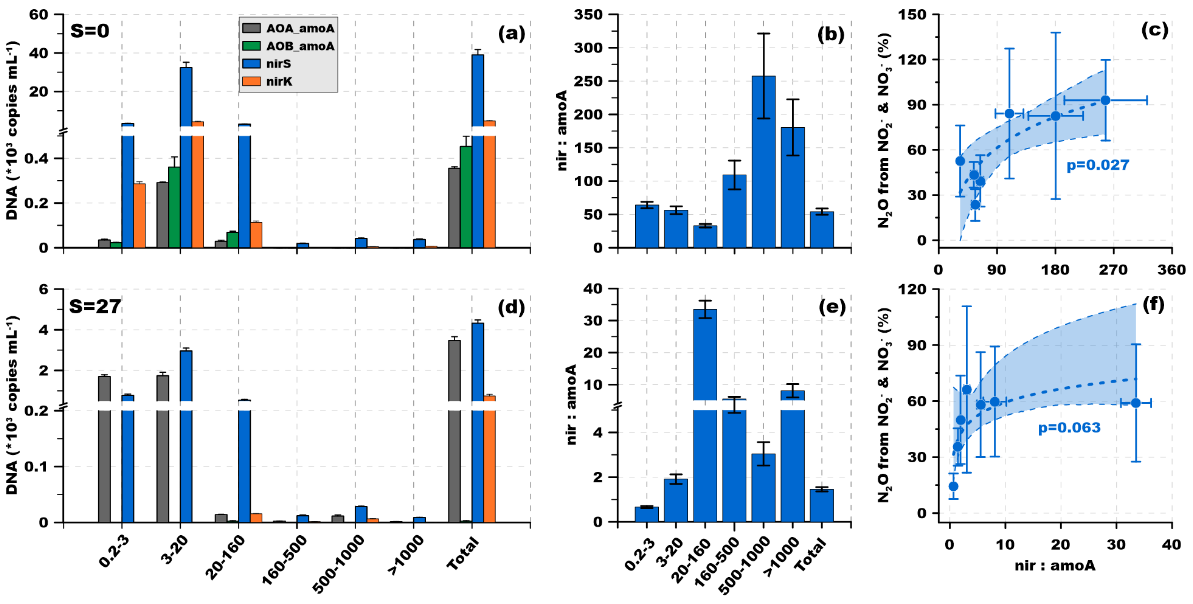

Supplementary Fig. 11 Functional genes (archaeal *amo*A, bacterial *amo*A, bacterial *nir*S and bacterial *nir*K) distribution and the relationship between gene abundance and rate distributions in size-fractionated particles at two stations in the JLE (2018). a, d: gene abundances at each size; b, e: nir: amoA at each size; c, f: relationship between fractional contribution of N_2O produced via NO_x^- reduction: (*nir*: *amoA*) at the JL0 (a-c) and JL27 (d-f) stations. Errors bars for the functional gene abundances represent standard deviation of triplicate measurement from one single sample (n = 1); errors bars for the fractional N_2O production are propagated standard deviation on the rates derived from triplicate incubations.

Supplementary Figures and Tables

Supplementary Table 1 Statistics of nutrient and rate distribution in the investigated estuaries.

	PRE		JLE		CJE	
	**Range**	**Median**	**Range**	**Median**	**Range**	**Median**
Temperature (°C)	19.9-31.4	22.3	28.5-31.4	28.8	9.2-29.1	21.4
DO (μmol L^{-1})	16-228	145	118-206	147	43-342	183
DO saturation (%)	7-105	62	53-105	69	20-137	87
NH_4^+ (μmol L^{-1})	0.4-393.7	8.1	6.5-33.5	12.4	0.1-0.7	0.4
NO_2^- (μmol L^{-1})	1.1-27.7	12.6	6.3-33.9	20.6	0.01-2.3	0.4
NO_3^- (μmol L^{-1})	1.2-294.5	109.3	22.9-235.7	118.2	0.04-40.1	7.3
DIN (μmol L^{-1})	4.1-556.3	130.2	13.6-276.5	152.9	0.4-25.7	8.1
TSM (mg L^{-1})	10-108	36	18-180	31	1-28	5
PN (μmol L^{-1})	5.4-24.7	11.3	3.7-15.5	7.5	0.7-19.5	1.5
PN: TSM (%)	0.1-1.5	0.5	0.1-0.6	0.3	0.1-6.9	0.7
N_2O (nmol L^{-1})	8.0-653.6	47.1	12.3-146.3	56.2	8.7-17.9	13.2
N_2O saturation (%)	106-6760	575	189-2084	786	118-229	175
N_2O flux (μmol m^{-2} d^{-1})	1.3-1592.8	26.8	8.9-228.0	75.7	4.0-14.7	10.7
NH_3 oxidation (μmol L^{-1} d^{-1})	0.1-20.9	1.0	0.2-22.1	0.6	0.0-0.1	0.03
NO_2^- oxidation (μmol L^{-1} d^{-1})	0.2-36.1	0.65	0.01-0.9	0.04	0.0-0.1	0.02
NO_3^- reduction (μmol L^{-1} d^{-1})	0.0-6.9	0.2	0.1-3.9	0.8	0.0-0.02	0.002
N_2O from NH_3 (nmol N L^{-1} d^{-1})	0.1-22.6	0.8	0.2-21.5	1.3	0.0-0.3	0.02
N_2O from NO_2^-(nmol N L^{-1} d^{-1})	0.1-29.8	0.3	0.1-7.3	0.4	0.0-0.5	0.02
N_2O from NO_3^-(nmol N L^{-1} d^{-1})	0.0-43.6	0.3	0.2-29.3	2.1	0.0-1.8	0.01
Total N_2O (nmol N L^{-1} d^{-1})	0.3-91.7	1.5	0.4-58.1	3.7	0.1-2.5	0.05
Fractional N_2O from NH_3 oxidation (%)	1.7-70.6	48.6	32.4-47.8	36.1	9.5-56.7	29.9
Fractional N_2O from NO_x^- oxidation (%)	29.4-98.3	51.4	52.2-67.6	63.9	43.3-90.5	70.1

Supplementary Table 2 Overview of sampling station information and experiments and measurements conducted at each station.

Cruise	Region	Station	Latitude (°N)	Longitude (°E)	Sample information	On-board incubation
2013, Nov.	PRE	P01	23.0737	113.4673	Nutrients, N_2O, PN, DO, TSM	Yes
2013, Nov.	PRE	P02	23.0544	113.2796	Nutrients, N_2O, PN, DO, TSM	Yes
2013, Nov.	PRE	P03	23.0737	113.4673	Nutrients, N_2O, PN, DO, TSM	Yes
2013, Nov.	PRE	P05	22.9510	113.5513	Nutrients, N_2O, PN, DO, TSM	Yes
2013, Nov.	PRE	P06	22.8668	113.5747	Nutrients, N_2O, PN, DO, TSM	No
2013, Nov.	PRE	P07	22.7871	113.6294	Nutrients, N_2O, PN, DO, TSM	Yes
2013, Nov.	PRE	A01	22.7386	113.6551	Nutrients, N_2O, PN, DO, TSM	Yes
2013, Nov.	PRE	A02	22.6854	113.7033	Nutrients, N_2O, PN, DO, TSM	No
2013, Nov.	PRE	A03	22.6060	113.7084	Nutrients, N_2O, PN, DO, TSM	Yes
2013, Nov.	PRE	A04	22.5218	113.7456	Nutrients, N_2O, PN, DO, TSM	No
2013, Nov.	PRE	A05	22.4547	113.7629	Nutrients, N_2O, PN, DO, TSM	No
2013, Nov.	PRE	A06	22.3963	113.7725	Nutrients, N_2O, PN, DO, TSM	Yes
2013, Nov.	PRE	A07	22.3206	113.8126	Nutrients, N_2O, PN, DO, TSM	No
2013, Nov.	PRE	A08	22.2566	113.8207	Nutrients, N_2O, PN, DO, TSM	Yes
2013, Nov.	PRE	A09	22.1997	113.8170	Nutrients, N_2O, PN, DO, TSM	No
2015, April	CJE	P1	31.5000	123.0000	Nutrients, N_2O, PN, DO	Yes
2015, April	CJE	C3	31.9967	124.0000	Nutrients, N_2O, PN, DO	Yes
2015, April	CJE	C5	33.9983	123.9967	Nutrients, N_2O, PN, DO	Yes
2016, Sep.	JLE	J1	24.3960	117.9203	Nutrients, N_2O, PN, DO, TSM	Yes
2016, Sep	JLE	J2	24.4153	117.8725	Nutrients, N_2O, PN, DO, TSM	No
2016, Sep	JLE	J3	24.4127	117.8801	Nutrients, N_2O, PN, DO, TSM	No
2016, Sep	JLE	J4	24.4109	117.8822	Nutrients, N_2O, PN, DO, TSM	No
2016, Sep	JLE	J5	24.4072	117.8879	Nutrients, N_2O, PN, DO, TSM	No
2016, Sep	JLE	J6	24.4040	117.8970	Nutrients, N_2O, PN, DO, TSM	Yes
2016, Sep	JLE	J7	24.4040	117.8970	Nutrients, N_2O, PN, DO, TSM	No
2016, Sep	JLE	J8	24.4016	117.9050	Nutrients, N_2O, PN, DO, TSM	No
2016, Sep	JLE	J9	24.3980	117.9136	Nutrients, N_2O, PN, DO, TSM	No
2016, Sep	JLE	J10	24.3969	117.9215	Nutrients, N_2O, PN, DO, TSM	No
2016, Sep	JLE	J11	24.3970	117.9260	Nutrients, N_2O, PN, DO, TSM	Yes
2016, Sep	JLE	J12	24.3992	117.9301	Nutrients, N_2O, PN, DO, TSM	No
2016, Sep	JLE	J13	24.4009	117.9287	Nutrients, N_2O, PN, DO, TSM	Yes
2016, Sep	JLE	J14	24.4014	117.9297	Nutrients, N_2O, PN, DO, TSM	No
2016, Sep	JLE	J15	24.4127	117.9417	Nutrients, N_2O, PN, DO, TSM	No
2016, Sep	JLE	J16	24.4019	117.9503	Nutrients, N_2O, PN, DO, TSM	No
2016, Sep	JLE	J17	24.4003	117.9498	Nutrients, N_2O, PN, DO, TSM	Yes
2016, Sep	JLE	J18	24.4011	117.9574	Nutrients, N_2O, PN, DO, TSM	No
2016, Sep	JLE	J19	24.4148	118.0017	Nutrients, N_2O, PN, DO, TSM	No
2016, Sep	JLE	J20	24.4181	118.0109	Nutrients, N_2O, PN, DO, TSM	No
2016, Sep	JLE	J21	24.4186	118.0163	Nutrients, N_2O, PN, DO, TSM	No
2016, Sep	JLE	J22	24.4189	118.0298	Nutrients, N_2O, PN, DO, TSM	Yes
2016, Sep	JLE	J23	24.4146	118.0555	Nutrients, N_2O, PN, DO, TSM	No
2016, Sep	JLE	J24	24.4075	118.0715	Nutrients, N_2O, PN, DO, TSM	No
2016, Sep	JLE	J25	24.3915	118.0854	Nutrients, N_2O, PN, DO, TSM	No
2016, Sep	JLE	J26	24.3759	118.1020	Nutrients, N_2O, PN, DO, TSM	Yes
2017, Aug.	CJE	S1	25.4174	119.9860	Nutrients, N_2O, PN, DO, TSM	Yes
2017, Aug.	CJE	S3	28.0008	121.7835	Nutrients, N_2O, PN, DO, TSM	Yes
2017, Aug.	CJE	A1	29.7125	122.5368	Nutrients, N_2O, PN, DO, TSM	Yes
2017, Aug.	CJE	E3	30.5106	123.3227	Nutrients, N_2O, PN, DO, TSM	Yes
2017, Aug.	CJE	B7	30.7898	123.1973	Nutrients, N_2O, PN, DO, TSM	Yes
2017, Aug.	CJE	B5	30.9530	122.9557	Nutrients, N_2O, PN, DO, TSM	Yes
2017, Aug.	CJE	B3	31.0608	122.3724	Nutrients, N_2O, PN, DO, TSM	No

2017, Aug.	CJE	F4	30.3660	122.7584	Nutrients, N_2O, PN, DO, TSM	No
2017, Aug.	CJE	A3	29.6618	122.8292	Nutrients, N_2O, PN, DO, TSM	No
2017, Aug.	CJE	S4	28.7373	122.0034	Nutrients, N_2O, PN, DO, TSM	No
2018, July	JLE	JL0	24.4250	117.8383	Nutrients, N_2O, PN, DO, TSM	Yes
2018, July	JLE	JL27	24.4015	118.0673	Nutrients, N_2O, PN, DO, TSM	Yes
2020, July	PRE	A1	22.8187	113.592	Nutrients, N_2O, DO	Yes
2020, July	PRE	A8	22.2031	113.8028	Nutrients, N_2O, DO	Yes
2020, July	PRE	A9	22.0264	113.8214	Nutrients, N_2O, DO	Yes
2020, July	PRE	A10	22.0841	113.7615	Nutrients, N_2O, DO	Yes

Supplementary Table 3 Overview of isotope labeling incubation experiments.

Cruise	Station & depth	Tracer (μmol L^{-1})			^{15}N (%)			Timepoints (hour)	Number of incubations
		NH_4^+	NO_2^-	NO_3^-	NH_4^+	NO_2^-	NO_3^-		
2013-PRE	P01-3m	20	2	20	4	9	11	0, 3, 6, 12, 24	Triplicates
2013-PRE	P02-5m	10	2	20	15	7	10	0, 3, 6, 12, 24	Triplicates
2013-PRE	P03-1m	2	2	20	25	7	7	0, 3, 6, 12, 24	Triplicates
2013-PRE	P03-5m	2	2	20	25	7	7	0, 3, 6, 12, 24	Triplicates
2013-PRE	P05-1m	2	2	20	33	17	9	0, 3, 6, 12, 24	Triplicates
2013-PRE	P05-6m	2	2	20	25	17	9	0, 3, 6, 12, 24	Triplicates
2013-PRE	P07-1m	1	2	20	8	9	7	0, 3, 6, 12, 24	Triplicates
2013-PRE	P07-7m	1	2	20	17	9	9	0, 3, 6, 12, 24	Triplicates
2013-PRE	A01-1m	2	2	20	15	15	11	0, 3, 6, 12, 24	Triplicates
2013-PRE	A01-15m	2	2	20	29	11	17	0, 3, 6, 12, 24	Triplicates
2013-PRE	A03-1m	1	2	10	12	13	7	0, 3, 6, 12, 24	Triplicates
2013-PRE	A03-5m	1	2	10	12	13	10	0, 3, 6, 12, 24	Triplicates
2013-PRE	A06-1m	0.5	0.5	2	12	3	4	0, 3, 6, 12, 24	Triplicates
2013-PRE	A06-6m	0.5	0.5	2	23	4	3	0, 3, 6, 12, 24	Triplicates
2013-PRE	A08-1m	0.5	0.5	2	25	9	7	0, 3, 6, 12, 24	Triplicates
2013-PRE	A08-11m	0.5	0.5	2	33	5	7	0, 3, 6, 12, 24	Triplicates
2015-ECS	P1-5m	0.3	0.3	0.5	56	44	3	0, 12, 24	Duplicates
2015-ECS	P1-10m	0.3	0.3	0.5	63	51	5	0, 12, 24	Duplicates
2015-ECS	P1-20m	0.3	0.3	0.5	67	47	5	0, 12, 24	Duplicates
2015-ECS	P1-30m	0.3	0.3	0.5	73	47	5	0, 12, 24	Duplicates
2015-ECS	C3-5m	0.3	0.3	0.5	57	40	8	0, 12, 24	Duplicates
2015-ECS	C3-15m	0.3	0.3	0.5	58	40	8	0, 12, 24	Duplicates
2015-ECS	C3-30m	0.3	0.3	0.5	56	41	8	0, 12, 24	Duplicates
2015-ECS	C5-5m	0.3	0.3	0.5	49	93	92	0, 12, 24	Duplicates
2015-ECS	C5-25m	0.3	0.3	0.5	64	96	63	0, 12, 24	Duplicates
2015-ECS	C5-33m	0.3	0.3	0.5	48	54	8	0, 12, 24	Duplicates
2015-ECS	C5-50m	0.3	0.3	0.5	70	76	4	0, 12, 24	Duplicates
2015-ECS	C5-60m	0.3	0.3	0.5	69	72	4	0, 12, 24	Duplicates
2015-ECS	C5-70m	0.3	0.3	0.5	68	77	4	0, 12, 24	Duplicates
2016-JLE	J1-2m	5	5	10	13	13	4	0, 6, 12	Triplicates
2016-JLE	J6-2m	5	5	10	33	33	6	0, 6, 12	Triplicates
2016-JLE	J11-2m	2	2	5	14	14	4	0, 6, 12	Triplicates
2016-JLE	J13-2m	2	2	5	13	13	5	0, 6, 12	Triplicates
2016-JLE	J17-2m	2	2	5	13	12	7	0, 6, 12	Triplicates
2016-JLE	J22-2m	2	2	5	13	16	9	0, 6, 12	Triplicates
2016-JLE	J26-2m	2	2	5	18	24	18	0, 6, 12	Triplicates
2017-CJE	S1-15m	0.5	0.5	1	53	36	24	0, 6, 12	Triplicates
2017-CJE	S3-25m	0.5	0.5	1	48	24	20	0, 6, 12	Triplicates
2017-CJE	A1-25m	0.5	0.5	1	52	50	6	0, 6, 12	Triplicates
2017-CJE	E3-25m	0.5	0.5	1	44	58	21	0, 6, 12	Triplicates
2017-CJE	F4-22m	0.5	0.5	1	45	32	6	0, 6, 12	Triplicates
2017-CJE	B5-10m	0.5	0.5	1	49	62	6	0, 6, 12	Triplicates
2018-JLE	JL0-2m	10	10	20	38	30	16	0, 3, 6	Triplicates
2018-JLE	JL27-2m	2	2	10	34	26	28	0, 3, 6	Triplicates
2020-PRE	A1-7m	2	2	12	52	43	9	0, 6, 12	Triplicates
2020-PRE	A8-5m	1	1	8	53	16	16	0, 6, 12	Triplicates
2020-PRE	A9-15m	1	1	4	59	31	76	0, 6, 12	Triplicates
2020-PRE	A10-8m	1	1	4	53	45	24	0, 6, 12	Triplicates

Supplementary Table 4 Comparison of *in-situ* N_2O production in the water column to air-sea N_2O flux and sedimentary N_2O production. The depth-integrated rate (from surface to bottom) was derived using trapezoidal extrapolation in stations where multiple depths were sampled. Note we excluded those stations where only one depth was sampled because the vertical resolution was too low to derive a reliable depth-integrated rate. The sedimentary N_2O production rate was derived as the average N_2O production rate from the intact core incubation from the literature.

**Area**	**Station**	**WC N_2O rate[a] ($\mu mol\ m^{-2}\ d^{-1}$)**	**Flux ($\mu mol\ m^{-2}\ d^{-1}$)**	**WC N_2O rate: flux (%)**	**SD N_2O rate[b] ($\mu mol\ m^{-2}\ d^{-1}$)**	**WC N_2O rate: SD N_2O rate (%)**
CJE	P1	1.5±0.8	14.7	10.3±5.6	2.6±2.5 (ref. 2)	57.9±31.4
CJE	C5	0.8±0.1	13.7	5.5±0.8	2.6±2.5 (ref. 2)	28.8±3.9
CJE	C3	0.5±0.2	14.6	3.7±1.3	2.6±2.5 (ref. 2)	20.5±7.1
PRE	A08	2.6±0.5	2.3	113.8±22.0	30.9±8.9 (ref. 3)	8.4±1.6
PRE	A06	2.4±1.2	7.2	33.6±5.2	30.9±8.9 (ref. 3)	7.8±1.2
PRE	A03	4.3±1.2	26.1	16.5±4.5	30.9±8.9 (ref. 3)	13.9±3.8
PRE	A01	16.6±6.2	32.2	51.5±19.3	30.9±8.9 (ref. 3)	53.6±20.0
PRE	P07	5.4±1.8	26.8	20.1±6.8	30.9±8.9 (ref. 3)	17.4±5.9
PRE	P05	10.3±5.4	53.5	19.3±10.0	30.9±8.9 (ref. 3)	33.3±17.3
PRE	P03	62.2±8.2	126.1	49.4±6.5	30.9±8.9 (ref. 3)	201.2±26.5

a: WC N_2O rate is the depth-integrated (surface to bottom) N_2O production rate of the water column.

b: SD N_2O rate is the sedimentary N_2O production rate measured using intact core.

Supplementary Table 5 Primer sets for qPCR of the four functional genes.

Gene	Reaction	Primer	Primer sequence 5’-3’	References
Archaeal	qPCR	Arch-amoAFA	GGGGTTTCTACTGGTGGT	(4)
*amo*A		Arch-amoAR	GCGGCCATCCATCTGTATGT	(4)
Bacterial	qPCR	amoA-1F	GGGGTTTCTACTGGTGGT	(5)
*amo*A		amoA-r New	CCCCTCBGSAAAVCCTTCTTC	(6)
*nir*K	qPCR	nirK876	ATYGGCGGVCAYGGCGA	(7)
		nirK1040	GCCTCGATCAGRTTRTGG	(7)
*nir*S	qPCR	nirS-1F	CCTAYTGGCCGCCRCART	(8)
		nirS-3R	GCCGCCGTCRTGVAGGAA	(8)

Supplementary Notes

Supplementary Note 1

Both NO_2^- and NO_3^- can be used as substrates by various denitrifiers, and the NO_2^- produced via NO_3^- reduction could exchange with the ambient NO_2^-, leading to potential double counting of N_2O production if we sum the $^{15}NO_2^-$ and $^{15}NO_3^-$ labeling incubations (i.e., it is likely that part of the N_2O production from $^{15}NO_2^-$ labeling incubation was sourced from the same pathway in the $^{15}NO_3^-$ labeling incubation). Currently, we are unable to quantitatively estimate the potential overlap of N_2O from NO_2^- and NO_3^-. Therefore, we compared the total N_2O production from $^{15}NO_2^-$ and $^{15}NO_3^-$ to N_2O production from $^{15}NO_3^-$ alone. The latter represents the minimal estimate of N_2O production via the reductive pathway, and was not much less than the total N_2O production rate, indicating N_2O from NO_2^- reduction was not the primary source of N_2O in our study area (Supplementary Fig. 6a). Comparing N_2O production from NH_4^+ vs NO_3^- alone, the fractional contribution of N_2O by ammonia oxidation increased with the most prominent change observed at the high salinity water, where both NH_4^+ and NO_3^- were low and the NO_2^- contribution to DIN pool was highest. Nevertheless, this conservative estimation demonstrated the NO_3^- reduction alone still contributed to a substantial fraction of N_2O (median value: 34, 58, 44% in the PRE, JLE and CJE, respectively), reinforcing a key role of denitrification to N_2O production in these oxic waters (Supplementary Fig. 6b).

Supplementary Note 2

The recovery efficiency of the particulate matter was assessed by comparing *in-situ* PN and POC concentrations with the summed concentrations of all sizes. The total recovered POC and PN were comparable with the *in-situ* concentrations at the upstream station JL0 (>95%); and were slightly lower than the *in-situ* concentrations at the lower estuarine station JL27 (>80%), indicating that our methods resulted in efficient recovery of particulate material (Supplementary Fig. 8 a-d). POC and PN concentrations in each size were consistent with the TSM distribution, i.e., the POC and PN were concentrated at the 3-20 μm size at the upstream station, and were mainly distributed at the 0.2-3 and 3-20 μm size at the downstream station. Similarly, the impacts of size-fractionation manipulation on the N conversion rates were examined by comparing the bulk rates (the rate measured using the *in-situ* water without manipulation) with the sum of size-fractionated rates. The summed rates of all size-fractionated

incubations were close to the bulk rates for ammonia oxidation (123% and 89% at the upstream and downstream stations, respectively); however, the summed rates of NO_3^- reduction to NO_2^- was lower than the bulk NO_3^- reduction rate (Supplementary Fig. 8e-h). This difference may indicate a loss of certain microbial activities during the size-fractionation manipulation due to the stress caused by the filtration during particle retrieval for the anaerobic NO_3^- reduction processes.

Supplementary References

1. Schlitzer, Reiner, Ocean Data View, https://odv.awi.de, (2021).
2. Tan, E. et al. Organic matter decomposition sustains sedimentary nitrogen loss in the Pearl River Estuary, China. *Sci. Total. Environ.* **648,** 508-517 (2019).
3. Tan, E et al. Quantitatively deciphering the roles of sediment nitrogen removal in environmental and climatic feedbacks in two subtropical estuaries. *Water Res.* **224,** 119121 (2022).
4. Beman, J. M., Popp, B. N., & Francis, C. A. Molecular and biogeochemical evidence for ammonia oxidation by marine Crenarchaeota in the Gulf of California. *ISME J.* **2,** 429-441 (2008).
5. Rotthauwe, J. H., Witzel, K. P. & Liesack, W. The ammonia monooxygenase structural gene amoA as a functional marker: molecular fine-scale analysis of natural ammonia-oxidizing populations. *Appl. Environ. Microbiol.* **63,** 4704-4712 (1997).
6. Hornek, R. et al. Primers containing universal bases reduce multiple *amoA* gene specific DGGE band patterns when analysing the diversity of beta-ammonia oxidizers in the environment. *J. Microbiol. Methods.*, **66,** 147–155 (2006).
7. Henry, S. et al. Quantification of denitrifying bacteria in soils by *nirK* gene targeted real-time PCR. *J. Microbiol. Methods.*, **59**, 327-335 (2004).
8. Braker, G., Fesefeldt, A. & Witzel, K. P. Development of PCR primer systems for amplification of nitrite reductase genes (*nirK* and *nirS*) to detect denitrifying bacteria in environmental samples. *Appl. Environ. Microbiol.* **64**, 3769-3775 (1998).
