# Supplementary material for: Particle-associated denitrification is the primary source of N2O in oxic coastal waters
Source: Nat Commun. 2023 Dec 13;14:8280. doi: 10.1038/s41467-023-43997-3 (PMC10719265; doi:10.1038/s41467-023-43997-3)
Supplement: Supplementary file 3 — Reporting Summary [file 41467_2023_43997_MOESM3_ESM.pdf]

nature portfolio

Corresponding author(s): Xianhui S. Wan

Last updated by author(s): Nov 13, 2023

# Reporting Summary

Nature Portfolio wishes to improve the reproducibility of the work that we publish. This form provides structure for consistency and transparency in reporting. For further information on Nature Portfolio policies, see our Editorial Policies and the Editorial Policy Checklist.

## Statistics

For all statistical analyses, confirm that the following items are present in the figure legend, table legend, main text, or Methods section.

| n/a | Confirmed | |
|---|---|---|
| ☐ | ☒ | The exact sample size (*n*) for each experimental group/condition, given as a discrete number and unit of measurement |
| ☐ | ☒ | A statement on whether measurements were taken from distinct samples or whether the same sample was measured repeatedly |
| ☐ | ☒ | The statistical test(s) used AND whether they are one- or two-sided<br>*Only common tests should be described solely by name; describe more complex techniques in the Methods section.* |
| ☒ | ☐ | A description of all covariates tested |
| ☒ | ☐ | A description of any assumptions or corrections, such as tests of normality and adjustment for multiple comparisons |
| ☐ | ☒ | A full description of the statistical parameters including central tendency (e.g. means) or other basic estimates (e.g. regression coefficient) AND variation (e.g. standard deviation) or associated estimates of uncertainty (e.g. confidence intervals) |
| ☐ | ☒ | For null hypothesis testing, the test statistic (e.g. *F*, *t*, *r*) with confidence intervals, effect sizes, degrees of freedom and *P* value noted<br>*Give P values as exact values whenever suitable.* |
| ☒ | ☐ | For Bayesian analysis, information on the choice of priors and Markov chain Monte Carlo settings |
| ☒ | ☐ | For hierarchical and complex designs, identification of the appropriate level for tests and full reporting of outcomes |
| ☐ | ☒ | Estimates of effect sizes (e.g. Cohen's *d*, Pearson's *r*), indicating how they were calculated |

*Our web collection on statistics for biologists contains articles on many of the points above.*

## Software and code

Policy information about availability of computer code

| | |
|---|---|
| Data collection | The nitrogen isotope data were collected by 'GC-IRMS software Isodat version 3.0, Thermo Fisher Scientific'. |
| Data analysis | The statistical analyses were performed using SPSS Statistics 26. |

For manuscripts utilizing custom algorithms or software that are central to the research but not yet described in published literature, software must be made available to editors and reviewers. We strongly encourage code deposition in a community repository (e.g. GitHub). See the Nature Portfolio guidelines for submitting code & software for further information.

## Data

Policy information about availability of data

All manuscripts must include a data availability statement. This statement should provide the following information, where applicable:

- Accession codes, unique identifiers, or web links for publicly available datasets
- A description of any restrictions on data availability
- For clinical datasets or third party data, please ensure that the statement adheres to our policy

All data needed to evaluate the conclusions in the paper are deposited in Zenodo database that can be accessed through https://doi.org/10.5281/zenodo.8092113

# Research involving human participants, their data, or biological material

Policy information about studies with human participants or human data. See also policy information about sex, gender (identity/presentation), and sexual orientation and race, ethnicity and racism.

| | |
|---|---|
| Reporting on sex and gender | No human participants were involved in this study. |
| Reporting on race, ethnicity, or other socially relevant groupings | No human participants were involved in this study. |
| Population characteristics | No human participants were involved in this study. |
| Recruitment | No human participants were involved in this study. |
| Ethics oversight | No human participants were involved in this study. |

Note that full information on the approval of the study protocol must also be provided in the manuscript.

# Field-specific reporting

Please select the one below that is the best fit for your research. If you are not sure, read the appropriate sections before making your selection.

☐ Life sciences ☐ Behavioural & social sciences ☒ Ecological, evolutionary & environmental sciences

For a reference copy of the document with all sections, see nature.com/documents/nr-reporting-summary-flat.pdf

# Ecological, evolutionary & environmental sciences study design

All studies must disclose on these points even when the disclosure is negative.

| | |
|---|---|
| Study description | The study was carried out to address the key knowledge gaps on the sources and rates of N2O production in coastal waters. We investigated the distribution and flux of N2O in three large estuaries and the adjacent coastal zones spanning large environmental gradients along the coast of China.<br>A total of 60 stations in the Changjiang Estuary, the East China Sea, the Jiulong Estuary, the Pearl River Estuary were sampled. 15N isotope labeling experiments were performed in 48 depths. These experiments were conducted to identify the N2O concentration, air-sea N2O flux, N2O production rates and pathways in the coastal zone of China.<br>These sampling regions provide a natural laboratory to study the effects of particle and substrate interactions on N cycling and the associated N2O generation processes, and can improve our understanding of the climate feedback to human perturbation in the global coastal ocean.<br>We found that multiple active microbial N2O production processes sustain the intense N2O flux in the highly eutrophied and turbid coastal zone, with particle-associated denitrification contributing a major part of N2O production. |
| Research sample | Samples were collected along the Pacific coastal zone of China during six research cruises conducted from 2013 to 2020 to the Changjiang Estuary and the adjacent East China Sea (2015 and 2017), the Jiulong Estuary and the adjacent Taiwan Strait (2016 and 2018), and the Pearl River Estuary (2013 and 2020) .<br>A total of 107 samples from various depths at 60 stations were collected for N2O distribution and flux measurements. On-board isotope labeling incubation experiments were performed at 48 depths for 31 selected stations that covering all the investigated estuaries.<br>These sampling stations provide a good representation of the turbid, heavily human-perturbed ecosystems, with large gradients of key environmental variables such as substrate, particles, DO, etc., making them an ideal research area to explore N2O dynamics. |
| Sampling strategy | A total of 107 samples (n=107 biologically independent samples) from various depths at 60 stations that spanned a wide range of temperature, salinity, DO, TSM, DIN (including NH4+, NO2- and NO3-) and organic matter gradients were collected for N2O distribution and flux measurements.<br>On-board isotope labeling incubation experiments were performed at 48 depths for 31 selected stations.<br>All incubations were carried out in triplicate (n=3 biologically independent samples), and time-course incubations were conducted to provide reliable rate measurements. |
| Data collection | Temperature and salinity were measured using the Seabird 911 CTD sensor package in the CJE and PRE cruises and were measured using YSI6600D sensors in the JLE cruises. DO concentration was measured using the Winkler titration method in the PRE (2013) and CJE (2015) cruises, and using the electrode microsensor (Unisense, Denmark) in the remaining cruises. Discrete seawater samples were collected using twelve 12-liter Niskin bottles mounted to the CTD rosette in the CJE, and PRE cruises, and were collected using a 5-liter Perspex hydrophore water sampler in the JLE cruises. Samples for chemical, biological and rate measurements were collected from the same casts. All the collected data are publicly accessible upon the publication of the present study.<br>Xianhui S. Wan, Bess B. Ward, and Shuh-Ji Kao collected and recorded the data. |
| Timing and spatial scale | Samples were collected along the Pacific coastal zone of China during six research cruises conducted from 2013 to 2020 to the Changjiang Estuary and the adjacent East China Sea (Apr. 2015 and Aug. 2017), the Jiulong Estuary and the adjacent Taiwan Strait (Sep. 2016 and July 2018), and the Pearl River Estuary (Nov. 2013 and July 2020) (Supplementary Fig. 1; Supplementary Table 1-3).<br>The intervals were determined based on time to complete each experiment and availability of time and materials on board the |

| | |
|---|---|
| | research vessel. |
| Data exclusions | No data was excluded. |
| Reproducibility | All experiments were conducted in the field using natural marine communities in six independent cruises. The active nitrogen transformation and N2O production processes were repeatedly observed in all cruises conducted in different seasons, years and estuaries. All the incubations were conducted using triplicate biologically independent samples. As such, the main results of our study should be reproducible.<br>Detailed methods of the experimental design and sampling are provided to ensure reproducibility of the approaches used. |
| Randomization | The incubation vials, sampling bottles were randomly allocated. |
| Blinding | Blinding has been used throughout the sample analysis and data acquisition processes. |

Did the study involve field work? ☒ Yes ☐ No

## Field work, collection and transport

| | |
|---|---|
| Field conditions | The cruises were carried out at different seasons. The weather was overall fine without any extreme weather conditions (e.g., temperature ranged from 10-35°C; no heavy rainfall). |
| Location | Changjiang Estuary and the adjacent East China Sea, the Jiulong Estuary and the adjacent Taiwan Strait, and the Pearl River Estuary. Water depth: <100m. |
| Access & import/export | The study was carried out in the coastal zone of China. All cruises were carried out in compliance with the local laws and regulations. No specific permit was needed for sample collection or shipping. |
| Disturbance | No disturbance was caused by this study. |

# Reporting for specific materials, systems and methods

We require information from authors about some types of materials, experimental systems and methods used in many studies. Here, indicate whether each material, system or method listed is relevant to your study. If you are not sure if a list item applies to your research, read the appropriate section before selecting a response.

### Materials & experimental systems

| n/a | Involved in the study |
|---|---|
| ☒ | ☐ Antibodies |
| ☒ | ☐ Eukaryotic cell lines |
| ☒ | ☐ Palaeontology and archaeology |
| ☒ | ☐ Animals and other organisms |
| ☒ | ☐ Clinical data |
| ☒ | ☐ Dual use research of concern |
| ☒ | ☐ Plants |

### Methods

| n/a | Involved in the study |
|---|---|
| ☒ | ☐ ChIP-seq |
| ☒ | ☐ Flow cytometry |
| ☒ | ☐ MRI-based neuroimaging |

## Plants

| | |
|---|---|
| Seed stocks | No plants were collected in the study. |
| Novel plant genotypes | No plants were collected in the study. |
| Authentication | No plants were collected in the study. |
